# Challenges of Classifying Stage B Heart Failure in a High-Risk Population

**DOI:** 10.3390/jcdd13010043

**Published:** 2026-01-12

**Authors:** Alice C. Cowley, Abhishek Dattani, Jian L. Yeo, Anna-Marie Marsh, Manjit Sian, Kelly S. Parke, Joanne Wormleighton, Anvesha Singh, Christopher P. Nelson, Gaurav S. Gulsin, Gerry P. McCann, Emer M. Brady

**Affiliations:** 1 Department of Cardiovascular Sciences, University of Leicester and the NIHR Biomedical Research Centre, Glenfield Hospital, Leicester LE3 9QP, UK; 2 British Heart Foundation (BHF) Leicester Centre of Research Excellence, Leicester LE3 9QP, UK

**Keywords:** type 2 diabetes, stage B heart failure, sex and ethnicity stratified thresholds

## Abstract

Background: Stage B heart failure (SBHF) increases the risk of symptomatic HF. Current guideline criteria for SBHF lack sex and ethnic thresholding and cardiac magnetic resonance (CMR) imaging cut-offs. We aimed to assess the prevalence of SBHF in a large cohort of people with type 2 diabetes (T2D) and healthy controls and propose a refined CMR definition for SBHF. Methods: Sex- and ethnic-specific thresholds for imaging criteria were derived from 373 healthy controls, who underwent CMR cine imaging. The current definition for SBHF and refined criteria was applied to our prospectively recruited and intensively phenotyped cohort of asymptomatic people with T2D and no evidence of cardiovascular disease. The prevalence of SBHF by different definitions was calculated and patient characteristics, including exercise capacity, were compared between those classified as Stage A vs. B HF. Finally, the refined criteria were also applied to the following two historical cohorts with symptomatic cardiovascular disease: severe aortic stenosis (AS *n* = 70) and HF with preserved ejection fraction (HFpEF *n* = 136). Results: A total of 423 people with T2D and a subset of 102 healthy controls who underwent echocardiography were prospectively recruited. Current guideline criteria classified 91% of those with T2D and 69% of the healthy controls as SBHF, suggesting a lack of specificity. Applying derived sex- and ethnicity-specific thresholds, combining echo and CMR measures, the prevalence of SBHF was reduced to 30% in those with T2D. Using the refined definition, those with Stage B HF had lower exercise capacity than those with Stage A HF (percentage predicted maximal oxygen consumption 81 ± 16% vs. 91 ± 20%, *p* < 0.001). Applying the refined definition to symptomatic AS and HFpEF participants classified 89% and 85% with abnormal cardiac remodelling. Conclusion: Current guideline criteria for SBHF are non-specific and likely of limited value in clinical practice. Refining these criteria with sex- and ethnic-specific thresholds may improve identification of those at risk of developing symptomatic disease. Further research is required to validate these criteria.

## 1. Background

Heart failure (HF) is a global health challenge associated with substantial individual suffering and socioeconomic burden, with prevalence expected to rise [1]. UK national data have reported 80% of people with HF are diagnosed in hospital [2], which confers a poorer prognosis than those diagnosed in primary care [3]. Therefore, it is imperative that patients at the highest risk of symptomatic HF are identified early, to implement preventative therapies, reduce the subsequent risk of HF, and improve prognosis. The American College of Cardiology (ACC)/American Heart Association (AHA)/Heart Failure Society of America (HFSA) have proposed a four-stage classification system for HF, ranging from those at risk of HF due to existing conditions such as type 2 diabetes (T2D) up to those with severe, symptomatic, and refractory disease, stages A to D [4]. Stage B HF (SBHF) refers to patients who remain asymptomatic yet have evidence of cardiovascular structural and functional abnormalities [4] and is associated with elevated risk of progressing to symptomatic HF [5]. The Olmsted County Heart Function Study reported 20% of the 826 participants with subclinical disease, Stage A or B HF, progressed in HF stage over four years [6]. However, imperatively 39% of those with SBHF at baseline regressed during the study period [6], underpinning the potential benefits of early diagnosis.

Among people with T2D, HF is the leading cause of hospitalisation [7], and when all other risk factors are controlled, the risk of HF in T2D persists [8]. Exercise capacity is a powerful prognostic marker of incident and advancing HF [9,10] and has consistently been shown to be reduced in people with T2D who have no known cardiovascular disease [11,12]. To date, the true prevalence of SBHF in T2D remains unknown, although previous work, employing different definitions, has reported a prevalence of subclinical disease between 48 and 58% in asymptomatic diabetes cohorts [13,14,15]. A single, unified definition for SBHF is required to allow us to identify those at elevated risk of overt HF, quantify prevalence, assess response to interventions, and allow comparisons between studies. The most recent ACC/AHA/HFSA guidelines on the management of HF give suggested criteria and thresholds to diagnose SBHF. These criteria are based on brain natriuretic peptides (BNPs or NT-proBNPs) and echocardiography, the first-line imaging modality. No criteria are proposed for cardiac magnetic resonance imaging (CMR), despite this being the gold-standard non-invasive technique for quantification of cardiac volumes, mass, function, and tissue characterisation [16]. Furthermore, SBHF criteria are not sex- or ethnic-specific despite known differences in cardiac geometry and function, which may lead to reduced sensitivity and/or specificity [17].

The aims of this study were to (1) assess the prevalence of SBHF using echocardiographic criteria in T2D and healthy controls, (2) refine the definition to include CMR measures, with sex and ethnic thresholding, and determine the relationship of SBHF, as classified by each definition, with aerobic exercise capacity, and (3) apply the proposed definition to a cohort of people with known symptomatic cardiovascular disease.

## 2. Methods

### 2.1. Study Design and Participants

**Figure 1:**
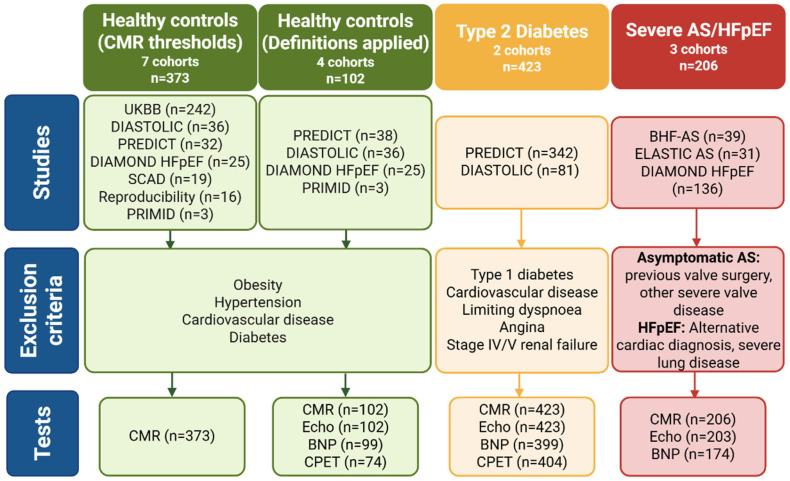
Study cohort grouped by disease status, describing exclusion criteria and study investigations analysed in each group. Created in biorender.com (accessed 7 January 2026).

This study included prospectively recruited participants grouped into the following three categories: (1) asymptomatic people with T2D, (2) healthy controls, and (3) participants with known cardiovascular disease (severe, symptomatic aortic stenosis (AS) or heart failure with preserved ejection fraction (HFpEF)) who were anticipated to have more cardiac remodelling (Figure 1).

Participants with asymptomatic T2D were recruited from the following two studies at our centre: (1) a prospective, cross-sectional observational study “Prevalence and Determinants of Subclinical Cardiovascular Dysfunction in Adults With Type 2 Diabetes Mellitus (PREDICT)” (NCT03132129) [18] and (2) the baseline data from a blinded end-point randomised controlled trial “Diabetes Interventional Assessment of Slimming or Training to Lessen Inconspicuous Cardiovascular Dysfunction (DIASTOLIC)” (NCT02590822) [19]. Participants with T2D were aged 18–75 years with a clinical diagnosis of T2D without signs or symptoms of cardiovascular disease. Exclusion criteria have been published previously [18].

The healthy control group was pooled from several studies using CMR cine imaging and had no symptoms or evidence of cardiovascular disease, hypertension, diabetes, or obesity [17,18,19,20,21,22,23]. A subset of this cohort also underwent serum natriuretic peptides, echocardiography, and cardiopulmonary exercise testing (CPET).

Finally, 2 cohorts of patients who demonstrate cardiac remodelling, typically with preserved ejection fraction, were as follows: severe, symptomatic AS [20,24] and HFpEF [21]. The AS cohort included patients aged 18–85 years with severe, isolated aortic valve disease; participants who had previous valve surgery or who were unable to exercise were excluded. The HFpEF cohort was aged >18 years, with radiological or clinical evidence of HF and an LV ejection fraction (EF) > 50%; participants with an alternative cardiac diagnosis (recent myocardial infarction, severe valve disease, cardiomyopathy, and constrictive pericarditis), severe lung disease, or a non-cardiovascular life expectancy of <6 months were excluded.

All studies were conducted in accordance with the Declaration of Helsinki and good clinical practice. Ethical approval was provided by the UK Health Research Authority Research Ethics Committee for each study and all participants provided written informed consent.

### 2.2. Assessments

Demographics, medical history, anthropometric measurements, and serum for HbA1c, kidney function, and natriuretic peptides were taken and analysed according to standard operating procedures in accredited University Hospitals of Leicester National Health Service Trust laboratories.

#### 2.2.1. Cardiovascular Magnetic Resonance (CMR)

A comprehensive CMR scan was performed on a 1.5- or 3-Tesla scanner (Siemens Skyra, Vida, or Aera scanner, Erlangen, Germany) using a standardised protocol. In brief, following cardiac localisers, standard balanced steady-state free precession cine images in 4-, 3-, and 2-chamber long-axis positions and short-axis slices, covering the whole left ventricle (LV), were taken [25]. Following the administration of a gadolinium-based contrast agent (0.15 mmol/kg Gadoteric acid, Dotarem, Guerbet, Villepinte, France), long- and short-axis late gadolinium enhancement (LGE) images were acquired using segmented phase-sensitive inversion recovery, free-breathing motion-corrected LGE techniques [26], or single-shot acquisitions, optimising individual participant image quality.

#### 2.2.2. Echocardiography

Transthoracic echocardiography was performed by one of two accredited operators, using an iE33b system with an S5-1 transducer (Philips Medical Systems, Best, The Netherlands) or a vivid E95 system with an 4Vc-D probe (GE Healthcare, Chicago, IL, USA). Images were acquired and reported as per the American Society of Echocardiography guidelines [27,28], and sonographers achieved excellent interobserver correlation.

#### 2.2.3. Cardiopulmonary Exercise Testing (CPET)

Exercise capacity was assessed in a temperature-controlled room using an incremental symptom-limited CPET (CASE Exercise Testing System, GE HealthCare, Chicago, IL, USA) with a bicycle ergometer (eBike Comfort, GE HealthCare). Calibration was performed prior to each assessment. A one-minute ramp protocol was used, with workload increments calculated based on participant age, sex, weight, and height [29]. Gas analysis was performed using a Ganshorn Powercube (Powercube-Ergo, GANSHORN Medizin Electronic GmbH, Niederlauer, Germany) and an appropriate post-processing software (Ganshorn LF8, GANSHORN Medizin Electronic GmbH, Niederlauer, Germany) using a 30 s rolling mean of breath-by-breath data. Percentage-predicted workload and maximal aerobic exercise capacity (peak VO_2_) were calculated using Wasserman–Hansen equations [30,31].

#### 2.2.4. Image Analysis

All CMR images were batch-analysed offline in a single centre by blinded observers (ACC, JY, KP, and GSG) who completed interobserver assessment and evaluation by an expert reader (GPM), using cvi42 5.10 (Circle Cardiovascular Imaging, Calgary, AB, Canada). Image quality was assessed prior to data analysis. Cardiac volumes were assessed in a semi-automated manner with manual correction as required; trabeculations and papillary muscles were included in the LV chamber volume and excluded from LV mass. Tissue tracking was used to quantify LV myocardial strain by contouring the endocardial and epicardial borders in the short- and long-axis cine images at end-diastole, presented as absolute values. LGE sequences were analysed qualitatively by an expert reader (GPM).

Echocardiography measurements were recorded if image quality enabled accurate quantification. If Simpson’s biplane LV EF was not quantifiable, a visual range was provided, with the median value of the range used for threshold application. Global longitudinal strain was measured using speckle tracking, analysis was semi-automated with manual correction, following the myocardium throughout the cardiac cycle (Philips Xcelera Qlab 9.0, Philips Healthcare, Andover, MA, USA).

### 2.3. Statistical Analysis

Data distribution was visually assessed using histograms and Q-Q plots. Continuous data were presented as mean (±standard deviation) where normally distributed, or median (25th to 75th percentile) as appropriate. Categorical variables were presented as count (percentage). Key clinical characteristics were described by diabetes status, and imaging parameters were indexed to height.

To determine if the clinical characteristics differed by HF definition, comparisons between those classified as Stages A and B HF were made using independent *t*-test or Mann–Whitney U test, as appropriate, and Chi-squared test for categorical variables. The relative difference in each criterion between Stage A and B HF for each definition was calculated (relative difference = (Stage B mean − Stage A mean)/Stage A mean). Finally, predicted exercise capacity was compared between Stage A and B HF by each definition, with adjustment for smoking status using analysis of covariance.

Statistical analysis was performed using StataCorp, 2023. Stata 18 Statistical Software. College Station, TX: StataCorp LLC [32].

#### 2.3.1. Derivation of SBHF Thresholds

The sex and ethnic thresholds for CMR were derived from the combined healthy control cohort. The cohort was split by sex and ethnicity giving rise to four groups (white males, non-white males, white females, and non-white females). Each group had a sample size greater than *n* = 40, the smallest sample size permitting the calculation of reference ranges using a parametric method for data with a Gaussian distribution [33]. For each quantitative CMR parameter, the threshold for abnormal was calculated as the mean plus or minus twice the standard deviation per group, depending on the direction of “disease”. The thresholds for echocardiographic parameters were guideline-driven [4,27,28].

#### 2.3.2. Refining the Definition of Stage B HF

Three definitions were applied to the cohort.

Definition 1: Current ACC/AHA/HFSA guidelines for the classification of SBHF [4] utilises echocardiography and serum natriuretic peptide levels (Supplementary Table S1). Briefly, these criteria include four domains covering cardiac morphology, LV systolic dysfunction, LV diastolic dysfunction, and brain natriuretic peptide, which includes a total of 12 components. To be classified as SBHF an individual must satisfy a single criterion across any domain. In this study, estimated pulmonary artery systolic pressure was not collected; therefore, 11 components were included.

Definition 2: Current ACC/AHA/HFSA guideline criteria but required to satisfy ≥2 criterion.

**Figure 2:**
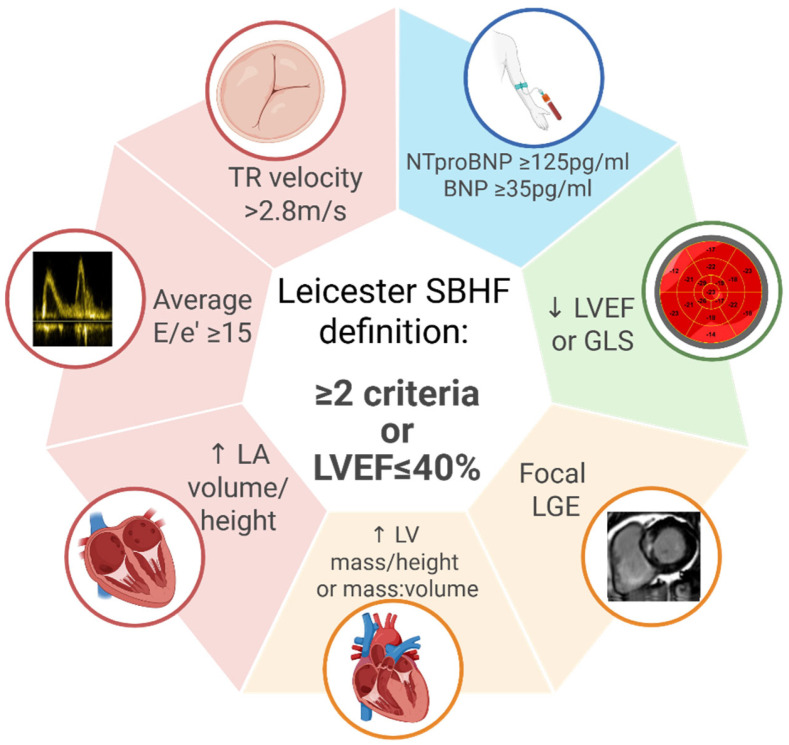
Definition 3—Stage B heart failure criteria using refined guideline components and sex- and ethnicity-specific cardiac magnetic resonance thresholds. Created in biorender.com (accessed 7 January 2026).

**Figure 3:**
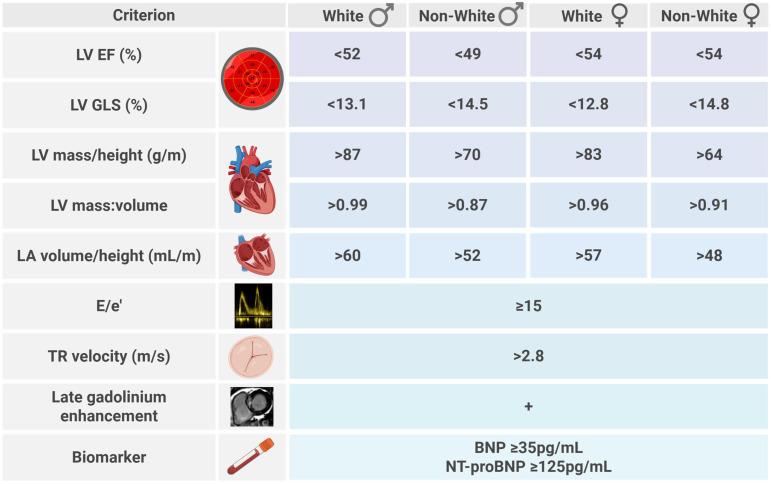
Thresholds for definition 3 Stage B heart failure, including sex- and ethnicity-specific cardiac magnetic resonance measures. Created in biorender.com (accessed 7 January 2026).

Definition 3: The four domains are preserved and echocardiographic measures for systolic function and cardiac remodelling replaced with CMR criteria (Figure 2), with the inclusion of sex and ethnic thresholds (Figure 3), alongside hallmark measures of diastolic function, tricuspid regurgitation velocity, and E/e’. Exploiting the advantages of CMR for tissue characterisation, late gadolinium enhancement was included to identify patients with focal fibrosis. In this definition, values were indexed to height, rather than body surface area, which underestimates the prevalence of LV hypertrophy in obesity. For the classification of SBHF, an individual was required to meet at least two criteria, with the caveat of HF with reduced EF ≤ 40% considered SBHF. To counter that more than one parameter was linked to measures of systolic function and LV hypertrophy, an “or” function was applied to these parameters. Finally, the highly non-specific e’ was excluded and the current evidence-base was reviewed to confirm the association of final components to symptomatic HF.

Definitions 1 and 2 were applied to the asymptomatic T2D and the sub-set of healthy controls who had echocardiography. Definition 3 (including CMR criteria) was applied to the asymptomatic T2D and symptomatic cardiovascular disease groups; it was not applied to the healthy controls given they were included in the derivation cohort for sex and ethnic thresholding.

## 3. Results

### 3.1. Study Cohorts

Clinical characteristics of the subset of healthy controls who had echocardiography (*n* = 102), the asymptomatic T2D (*n* = 423), severe, symptomatic AS (*n* = 70), and HFpEF (*n* = 136) cohorts are provided in Table 1. Within the asymptomatic T2D group, almost half (48%, *n* = 203) were prescribed angiotensin-converting enzyme inhibitors or angiotensin receptor blockers and 26% (*n* = 110) were taking a sodium-glucose co-transporter-2 inhibitor and/or glucagon-like peptide-1 receptor agonist. Half of the HFpEF cohort and 29% of the severe, symptomatic AS cohort also had diabetes; no participants in this group were taking sodium-glucose co-transporter-2 inhibitors or glucagon-like peptide-1 receptor agonists.

In the pooled healthy control group (*n* = 373) for thresholding CMR criteria with sex and ethnic stratification, the smallest group was non-white females, then non-white males, followed by white females, with the largest group being white males (*n* = 58, 91, 95, and 129, respectively). The cohort comprised middle-aged patients (57 ± 10 years). Anthropometric measures included: BMI 24.6 ± 2.7 kg/m^2^, systolic blood pressure (BP) 125 ± 14 mmHg, and heart rate 66 ± 10 bpm.

### 3.2. Prevalence of Stage B Heart Failure by Definition

**Figure 4:**
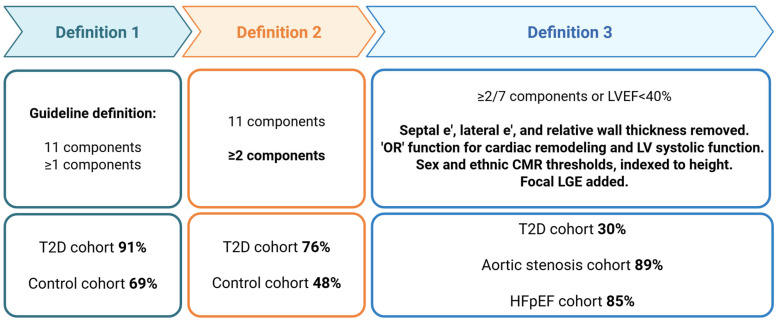
Prevalence of Stage B heart failure by each definition. Created in biorender.com (accessed 7 January 2026).

Definition 1 classified 91% (*n* = 386) of asymptomatic T2D and 69% (*n* = 70/102) of healthy controls with SBHF (Figure 4). In particular, high proportions of septal e’, lateral e’, and relative wall thickness were classed as abnormal in both cohorts (Supplementary Table S2 and the Supplementary Figure S1).

Definition 2 reduced the number classified as SBHF in both T2D and healthy control cohorts (76% (*n* = 322) and 48% (*n* = 49), respectively).

Definition 3 classified 30% (*n* = 128) of asymptomatic T2D with SBHF, 89% (*n* = 62) of severe, symptomatic AS, and 85% (*n* = 116) of HFpEF as having abnormal cardiac remodelling.

### 3.3. Comparison of Characteristics Between Stage A and B Heart Failure in the Type 2 Diabetes Cohort

Applying definitions 1 and 2, those classified as SBHF were more likely to be older adults, whereas the SBHF group using the CMR approach (definition 3) were significantly younger. Sex and glycosylated haemoglobin did not differ between Stages A and B HF across definitions. BMI and systolic BP were higher in SBHF for all definitions. Those classified as SBHF using definition 3 were more likely to have a history of smoking and be of non-white ethnicity (Table 2).

Applying definition 1, percentage-predicted workload was significantly greater in the SBHF group compared to the group classified as Stage A HF (*p* = 0.015), there was no difference in percentage-predicted peak VO_2_ (Table 2). Applying definition 2, exercise capacity parameters did not differ between Stage A and B HF. Finally, applying definition 3 showed a significantly lower exercise capacity in the SBHF group. Percentage-predicted workload and percentage-predicted peak VO_2_ (exercise metrics that account for age, sex, height, and weight) were both lower in the SBHF group, independent of smoking status (*p* < 0.001 for both).

The relative difference between Stage B and Stage A HF for each criterion by definition is provided in Table 3. For each definition, where a relative difference was observed between Stage A and B HF for a given criterion, it was almost exclusively in the direction of “disease”. For mass and volumes, the relative difference was greatest between Stage A and B HF when applying definition 3 (indexed values for left atrial volume (relative difference using definition 1, definition 2, and definition 3; 0.08, 0.00, and 0.47), LV end-diastolic volume (0.06, 0.00, 0.33), and LV mass (0.09, 0.07, 0.32)).

## 4. Discussion

In this prospective cohort study, over 90% of asymptomatic participants with T2D were classified as having SBHF by the current ACC/AHA/HFSA guideline criteria; however, when the same criteria were applied to our cohort of asymptomatic healthy volunteers, without risk factors for cardiovascular disease, over two thirds were also classified as SBHF. These findings suggest the current proposed criteria for defining SBHF are non-specific and likely to be of little value if used in routine clinical practice. Indeed, our findings suggest that a large proportion of people would be incorrectly assigned to SBHF and may undergo unnecessary investigations and treatment. The refined definition (definition 3) was applied to an asymptomatic group of T2D, where it identified 30% as having SBHF. Due to a lack of longitudinal data, the refined definition was also applied to our cohorts with established cardiovascular disease where it correctly classified 89% of people with severe, symptomatic AS and 85% of those with HFpEF as having abnormal cardiac remodelling.

Previous criteria applied in research to identify SBHF have been heterogenous (Supplementary Table S3) [13,14,15,34]. The only universal similarity between criteria that have been applied is the inclusion of a measure of LV hypertrophy, although thresholds differ between publications. In addition, there is a theme of combining measures that assess systolic and diastolic dysfunction alongside structural changes. Only one previous publication included raised biomarkers as part of the criteria to diagnose SBHF [34], despite the known association of raised natriuretic peptides to incident HF in disease-free individuals [35]. In addition, all previous attempts to identify SBHF have exclusively used echocardiography for imaging parameters rather than the non-invasive reference standard for quantification of cardiac volumes and ejection fraction, CMR imaging [16].

Moreover, the thresholds used to delineate abnormal measurements in previous studies are diverse and frequently lack an appreciation of the known differences in cardiac structure between ages, sexes [36], and ethnicities [17]. This is similar to guideline criteria where just one parameter is stratified by sex; furthermore, the origin of thresholds in these criteria is not elucidated, including those values that are not commonplace in clinical practice [4]. When reviewing the CMR literature for thresholds to apply to our cohort, we found data that were generally specific to a single ethnicity [36], reported confidence intervals rather than prediction intervals [37], demonstrated large between study heterogeneity where meta-analyses were performed [37], or included participants with hypertension, diabetes, and obesity in their cohorts [38]. Therefore, the application of these cut-offs for refining the current clinical definition would have been inappropriate in this context, and appreciating sex and ethnic differences in these measurements is a foremost strength of this work. The components from the ACC/AHA/HFSA guideline criteria that were included in the revised criteria (definition 3) were selected based on their known association to incident HF [35,39,40,41,42,43,44], HF hospitalisation [45,46], and raised filling pressures [28]. Stipulating that two or more abnormal thresholds were met to identify SBHF (with the exception of a significantly low LV EF in definition 3) was required to prevent misclassifying individuals with non-pathological, isolated abnormal measurements that lie outside the 95% confidence interval.

Increasing age is associated with an increased risk of HF [35]. Whilst it is important to acknowledge this, it is also important to ensure criteria are not simply differentiating those at greatest risk based on age by utilising age-dependent measures such as e’ [28,47], which was subsequently removed from definition 3 due to the high prevalence it displayed in the cohort. Additionally, cardiac function and morphology changes with increasing age [36]. Age-specific CMR thresholds were considered in definition 3 initially; however, minimal differences were observed between the deciles in our cohort and therefore these were removed in favour of larger, more statistically robust sex and ethnic groups.

When comparing clinical characteristics between Stage A and B HF across each definition, BP and BMI were consistently greater in the SBHF groups; this is not surprising given the known association of hypertension and obesity to incident HF [48]. Definition 3 shows the greatest absolute difference in BMI between Stage A and B HF, and the greatest relative difference in gold-standard CMR measures of mass and volume. CMR indices were purposely indexed to height rather than body surface area, which is widely used for echocardiography, to avoid falsely normalising measurements in overweight/obese individuals and reducing the sensitivity of thresholds [49], which is particularly poignant in a cohort of people with T2D.

Although definition 3 uses components from two imaging modalities, it is the only definition to differentiate Stages A and B HF by percentage-predicted workload and percentage-predicted peak VO_2_, an important prognostic marker in HF [9,10]. This finding is likely to reflect the added value of sex and ethnic thresholding, indexing to height where applicable and to the imaging accuracy of CMR, in this largely overweight and obese cohort. Furthermore, where the focus of classifying SBHF is to identify those most at risk of symptomatic disease, the difference in exercise capacity between Stage A and B HF observed in definition 3 is suggestive of a greater prognostic ability of this definition.

It is important to emphasise that we are not proposing that CMR is used first-line to detect SBHF in large-scale populations—we would need evidence that this is cost-effective; however, the purpose of the current analyses was to demonstrate limitations in the currently proposed criteria and the need for echocardiographic sex- and ethnic-specific normal ranges. We are currently exploring whether SBHF in T2D can be predicted accurately by developing a clinical risk score (NCT03132129).

Strengths of this work include the systematic, methodical approach that centred around current guidance and evidence. Furthermore, the definitions were applied to a large, multi-ethnic cohort that underwent a high degree of unified phenotyping and is representative of the local population. Sex and ethnicity are known modifiers of cardiac measurements [17,50]—accounting for these demographics is essential to prevent misclassification—and build on current guidelines where these differences are only considered for one of the twelve criteria. The components included in the refined definition are all associated with symptomatic HF and provide a multifaceted evaluation of HF. Finally, the use of gold-standard CMR imaging provides a robust and reproducible assessment of cardiovascular structure and function.

This work is limited by its cross-sectional nature and lack of outcome data. Applying this refined definition to a cohort with incident HF outcome data would enable comprehensive external validation and determine if its classification of SBHF correctly identifies those at increased risk of developing symptomatic disease. There is potential merit in applying the refined definition to other cohorts at risk of HF, i.e., obese or hypertensive; the previous literature has not explored the prevalence of SBHF in these groups and more widespread application may provide insight into the definitions’ generalisability. A limitation of definition 3 is that it necessitates two imaging modalities, which limits its use clinically; however, cardiac imaging in asymptomatic individuals is uncommon and therefore an imaging definition for SBHF will always be more applicable in research cohorts. CMR imaging was preferred for cardiac quantitation to utilise its increased accuracy and reproducibility, alongside imaging for focal fibrosis. Furthermore, whilst we derived sex- and ethnicity-specific reference ranges, we could only divide our cohort into white and non-white groups. Appreciating ethnic differences in structure and function is imperative in cardiac imaging research—applying ethnic-specific thresholds is a strength of this work; however, our cohort allowed for just two delineations, which lacks the ethnic granularity that is important in larger, more ethnically diverse cohorts.

## 5. Conclusions

When applying current ACC/AHA/HFSA Stage B HF criteria, almost all asymptomatic people with T2D and over two thirds of healthy volunteers with no cardiovascular risk factors are classed as having Stage B HF. These findings suggest that the current criteria, and particularly echocardiographic e’, are non-specific and need improvement. Refining these criteria with sex- and ethnic-specific thresholds may improve identification of those at risk of symptomatic disease, but further work is required to validate this work.

## Figures and Tables

**Table 1:** Clinical characteristics of study cohorts.

Clinical Characteristics	Healthy Controls (*n* = 102)	Type 2 Diabetes (*n* = 423)	Aortic Stenosis (*n* = 70)	HFpEF (*n* = 136)
Age, years	59 ± 11	61 ± 9	70 (62, 74)	74 (67, 78)
Male sex	49 (48%)	258 (61%)	53 (76%)	67 (49%)
White ethnicity	75 (74%)	311 (74%)	64 (91%)	114 (84%)
Current or ex-smoker	27 (26%)	187 (44%)	44 (63%)	72 (53%)
Hypertensive	-	240 (57%)	47 (67%)	123 (90%)
T2D duration (years)	-	10 (5, 21)	-	-
Statin	12 (12%)	292 (69%)	49 (72%)	86 (63%)
Body mass index (kg/m^2^)	24.5 ± 2.2	31.5 ± 6.2	29.9 ± 5.3	33.8 ± 7.0
Clinic systolic blood pressure (mmHg)	133 ± 22	137 ± 17	136 ± 22	145 ± 25
Heart rate (bpm)	65 ± 10	76 ± 12	67 ± 14	67 ± 14
Glycosylated haemoglobin (%)	-	7.3 ± 1.2	-	6.2 (5.7, 7.3)
eGFR (ml/min/1.73^2^)	87 ± 12	87 ± 15	75 ± 17	64 ± 21
Maximum workload (W) *	142 (100, 200)	115 (87, 150)	-	-
Peak VO_2_ (mL/Kg/min) *	25.5 ± 7.6	19.0 ± 5.2	-	-
% predicted workload *	124 ± 32	100 ± 25	-	-
% predicted VO_2_ Max *	97 ± 21	88 ± 20	-	-

eGFR, estimated glomerular filtration rate; T2D, type 2 diabetes. * *n* = 74 in healthy control cohort for cardiopulmonary exercise testing measures.

**Table 2:** Clinical characteristics of the T2D cohort by SBHF definition.

	Definition 1 Current ACC/AHA/HFSA			Definition 2 ≥2 ACC/AHA/HFSA Criteria			Definition 3 ≥2 Criteria or LVEF ≤ 40%		
	Stage A *n* = 37	Stage B *n* = 386	*p*	Stage A *n* = 101	Stage B *n* = 322	*p*	Stage A *n* = 295	Stage B *n* = 128	*p*
Age, years	58 ± 7	61 ± 9	**0.028**	58 ± 9	62 ± 8	**<0.001**	62 ± 8	59 ± 10	**0.002**
Male sex	25 (68%)	233 (60%)	0.391	59 (58%)	199 (62%)	0.543	172 (58%)	86 (67%)	0.085
White ethnicity	27 (73%)	284 (74%)	0.937	77 (76%)	234 (73%)	0.478	227 (77%)	84 (66%)	**0.015**
Current/ex-smoker	15 (41%)	172 (45%)	0.638	38 (38%)	149 (46%)	0.127	119 (40%)	68 (53%)	**0.015**
Hypertensive	20 (54%)	220 (57%)	0.730	52 (51%)	188 (58%)	0.222	161 (55%)	79 (62%)	0.173
T2D duration, years	6 (3, 11)	8 (5, 12)	0.274	5 (3, 10)	8 (5, 13)	**0.002**	8 (5, 13)	6 (4, 11)	0.067
BMI, Kg/m^2^	29.5 ± 5.6	31.7 ± 6.2	**0.036**	30.3 ± 6.5	31.9 ± 6.0	**0.019**	30.3 ± 5.5	34.2 ± 6.9	**<0.001**
Average SBP, mmHg	130 ± 13	137 ± 17	**0.013**	131 (15)	138 (17)	**<0.001**	134 (15)	143 ± 18	**<0.001**
HbA1c, %	7.5 ± 1.4	7.3 ± 1.1	0.242	7.2 ± 1.2	7.4 ± 1.1	0.211	7.3 ± 1.2	7.2 ± 1.1	0.360
Exercise capacity									
% predicted workload *	91 ± 19	101 ± 25	**0.015**	98 ± 25	101 ± 25	0.297	104 ± 26	93 ± 21	**<0.001**
% predicted VO_2_ Max *	83 ± 17	88 ± 20	0.102	88 ± 19	88 ± 20	0.794	91 ± 20	81 ± 16	**<0.001**

T2D, type 2 diabetes; SBP, systolic blood pressure; and HbA1c, glycosylated haemoglobin. * *p* values adjusted for smoking history.

**Table 3:** Imaging parameters and NT-proBNP in Stages A and B heart failure by each definition in the type 2 diabetes cohort.

	Definition 1 Current ACC/AHA/HFSA			Definition 2 ≥2 ACC/AHA/HFSA Criteria			Definition 3 ≥2 Criteria or LVEF ≤ 40%		
	Stage A *n* = 37	Stage B *n* = 386	RD	Stage A *n* = 101	Stage B *n* = 322	RD	Stage A *n* = 295	Stage B *n* = 128	RD
RWT	0.36 ± 0.04	0.48 ± 0.09	0.33	0.39 ± 0.06	0.50 ± 0.09	0.28	0.47 ± 0.09	0.48 ± 0.09	0.02
Septal e’	8.6 ± 1.1	6.6 ± 1.7	−0.23	8.1 ± 1.2	6.3 ± 1.6	−0.22	7.0 ± 1.6	6.1 ± 1.9	−0.13
Lateral e’	12.3 ± 1.6	9.1 ± 2.4	−0.26	11.6 ± 1.9	8.7 ± 2.3	−0.25	9.6 ± 2.3	9.0 ± 3.0	−0.06
Average E/e’	7.5 ± 1.3	9.1 ± 2.3	0.21	7.7 ± 1.7	9.4 ± 2.3	0.22	8.9 ± 2.0	9.2 ± 2.8	0.03
Max. TR, m/s	2.4 ± 0.2	2.3 ± 0.4	−0.04	2.3 ± 0.3	2.3 ± 0.4	0.00	2.3 ± 0.4	2.4 ± 0.4	0.04
LAVi, mL/m	39 ± 19	42 ± 17	0.08	41 ± 19	41 ± 17	0.00	36 ± 11	53 ± 22	0.47
LV EDVi, mL/m	81 ± 27	86 ± 28	0.06	86 ± 29	86 ± 28	0.00	78 ±19	104 ± 37	0.33
LVMi, g/m	69 ± 17	75 ± 22	0.09	71 ± 19	76 ± 22	0.07	68 ± 15	90 ± 25	0.32
LV mass: volume	0.88 ± 0.13	0.89 ± 0.14	0.01	0.86 ± 0.13	0.90 ± 0.14	0.05	0.88 ± 0.12	0.91 ± 0.17	0.03
GLS, %	16.7 ± 2.4	16.6 ± 2.5	−0.01	17.1 ± 2.3	16.5 ± 2.5	−0.04	16.7 ± 2.1	16.4 ± 3.3	−0.02
LV EF, %	67 ± 5	67 ± 7	0.00	66 ± 6	67 ± 7	0.02	67 ± 6	66 ± 8	−0.01
LGE	1 (3%)	84 (22%)	-	7 (7%)	78 (24%)	-	32 (11%)	53 (41%)	-
NT-proBNP (pg/mL)	37 (18–69)	42 (18–72)	0.14	37 (18–63)	43 (18–76)	0.16	42 (18–68)	41 (18–87)	−0.02

Indices shaded in grey were measured by cardiac magnetic resonance. RD, relative difference; RWT, relative wall thickness; TR, tricuspid regurgitation (recorded if measurable); LAVi, left atrial volume indexed to height; LV, left ventricle; EDVi, end-diastolic volume indexed to height; LVMi, left ventricular mass indexed to height; GLS, global longitudinal strain; EF, ejection fraction; LGE, late gadolinium enhancement; and NT-proBNP, N-terminal pro b-type natriuretic peptide.

## Data Availability

The datasets presented in this article are not currently available because the data are part of an ongoing study. Requests to access the datasets should be directed to Professor Gerry McCann.

## Supplementary Materials

The following supporting information can be downloaded at https://www.mdpi.com/article/10.3390/jcdd13010043/s1, Figure S1: Prevalence of each imaging criterion for SBHF definitions using their respective thresholds; Table S1: Suggested criteria for identifying cardiac structural, functional changes, and raised filling pressures, as per the ACC/AHA/HFSA guidance; Table S2: Prevalence of each criterion for SBHF definitions in people with type 2 diabetes and healthy volunteers, using their respective thresholds; Table S3: Stage B heart failure definitions previously applied in the literature [13,14,15,34].

## Abbreviations

ACC	American College of Cardiology
AHA	American Heart Association
AS	Aortic stenosis
BMI	Body mass index
BP	Blood pressure
CMR	Cardiac magnetic resonance
CPET	Cardiopulmonary exercise testing
EF	Ejection fraction
eGFR	Estimated glomerular filtration rate
HC	Healthy controls
HF	Heart failure
HFpEF	Heart failure with preserved ejection fraction
HFSA	Heart failure society of America
LGE	Late gadolinium enhancement
LV	Left ventricular
SBHF	Stage B heart failure
T2D	Type 2 diabetes
