# Supplementary material for: Challenges of Classifying Stage B Heart Failure in a High-Risk Population
Source: J Cardiovasc Dev Dis. 2026 Jan 12;13(1):43. doi: 10.3390/jcdd13010043 (PMC12841935; doi:10.3390/jcdd13010043)
Supplement: Supplementary file 1 [file jcdd-13-00043-s001.zip › jcdd-4030387-supplementary.pdf]

Supplemental tables

Table S1. Suggested criteria for identifying cardiac structural, functional changes and raised filling pressures, as per the ACC/AHA/HFSA guidance.

Morphology	LAVI ≥ 29ml/m^2<br>LVMI >95g/m^2 female or >115g/m^2 male<br>RWT > 0.42<br>LV wall thickness ≥12mm
LV systolic dysfunction	LVEF <50%<br>GLS <16%
LV diastolic dysfunction	Average E/e' ≥15 for raised filling pressures<br>Septal e' <7cm/s<br>Lateral e' <10cm/s.<br>TR velocity >2.8m/s<br>Estimated PA systolic pressure >35mmHg
Biomarker*	BNP ≥35pg/mL<br>NT-proBNP ≥125pg/mL

*To be taken in the context of the population being screened, usually higher cut-offs are suggested in chronic kidney disease, atrial fibrillation and older patients. BNP, brain natriuretic peptide; GLS, global longitudinal strain; LAVI, left atrial volume indexed; LV, left ventricular; LVEF, left ventricular ejection fraction; LVMI, left ventricular mass indexed; NT-proBNP, N-terminal pro b-type natriuretic peptide; PA, pulmonary artery; RWT, relative wall thickness; TR, tricuspid regurgitation.

Table S2. Prevalence of each criterion for SBHF definitions in people with type 2 diabetes and healthy volunteers, using their respective thresholds.

Criterion	Definition 1 + 2*		Definition 3		
	**T2D (n=423)**	**HV (n=102)**	**T2D (n=423)**	**AS (n=70)**	**HFpEF (n=136)**
↑ LAVi	49 (11.6%)	28 (27.5%)	72 (17.0%)	16 (22.9%)	63 (46.3%)
↑ LVMi	52 (12.3%)	12 (11.8%)	122 (28.8%)	54 (77.1%)	48 (35.3%)
↑ RWT	280 (66.2%)	40 (39.2%)	-	-	-
↑ LV wall thickness	189 (44.7%)	18 (17.7%)	-	-	-
Concentric remodelling	-	-	118 (27.9%)	50 (71.4%)	42 (30.9%)
↑ LVMi or concentric remodelling	-	-	205 (48.5%)	60 (85.7%)	65 (47.8%)
↓ LVEF	7 (1.7%)	2 (2.0%)	7 (1.7%)	3 (4.3%)	6 (4.41%)
↓ GLS	5 (1.2%)	3 (2.9%)	44 (10.4%)	28 (40.0%)	42 (30.2%)
↓ LVEF or ↓ GLS	-	-	44 (10.4%)	29 (41.4%)	43 (31.6%)
↑ E/e′	9 (2.1%)	1 (1.0%)	9 (2.1%)	19 (27.1%)	35 (25.7%)
↓ Septal e′	237 (56.0%)	34 (33.3%)	-	-	-
↓ Lateral e′	253 (59.8%)	36 (35.3%)	-	-	-
↑ TR velocity	3 (0.7%)	1 (1.0%)	3 (0.7%)	3 (4.3%)	13 (9.6%)
Late gadolinium enhancement (yes)	-	-	85 (20.1%)	48 (68.6%)	63 (46.3%)
↑ Natriuretic peptides	36 (8.5%)	12 (11.8%)	36 (8.5%)	36 (51.4%)	53 (39.0%)
≥ 1 criterion	386 (91.3%)	70 (68.6%)	-	-	-
≥ 2 criteria	322 (76.1%)	49 (48.0%)	128 (30.3%)	62 (88.6%)	116 (85.3%)

*The same thresholding was used for these two definitions therefore they are presented together. T2D, type 2 diabetes; HV, healthy volunteers; LAVi, left atrial volume indexed to height; LVMi, left ventricular mass indexed; RWT, relative wall thickness; LV, left ventricle; LVEF, left ventricular ejection fraction; GLS, global longitudinal strain; TR, tricuspid regurgitation (recorded if measurable).

Table S3. Stage B heart failure definitions previously applied in literature.

Publication	SBHF definitions (≥ one criteria required)	SBHF Prevalence
Kiencke et al. 2010<br>n=100<br>Diabetes outpatients with no known cardiovascular disease.(1)	**LV systolic dysfunction** - LV EF<45%, LV end-diastolic internal dimension index >3.2 cm/m² or LV end-diastolic volume index >102 mL/m².<br>**LV diastolic dysfunction** – assessed by E/A, delta E/A with Valsalva manoeuvre, E/e', E-wave deceleration time, A-wave duration and pulmonary venous flow measurements including peak systolic and diastolic flow velocities and duration of atrial flow reversal.<br>**LV hypertrophy** (≥131g/m² for men, ≥100g/m² for women).	48%
Wang et al. 2018<br>n=290<br>Asymptomatic T2D outpatients ≥65 years.(2)	**LV hypertrophy** (>115g/m² for men, >95g/m² for women).<br>**LA enlargement** >34ml/m².<br>**E/e'**>13.<br>**GLS** <16%.	58%
Oo et al. 2021<br>n=305<br>T2D patients with no known cardiovascular disease.(3)	**Diastolic dysfunction** as per American Society Echocardiography guidelines 2016.<br>**LV hypertrophy** (>115g/m² for men, >95g/m² for women).<br>**LA enlargement** >34ml/m².	57%
Mohebi et al. 2023<br>n=11,618<br>3 prospective observational community cohorts.(4)	**LV EF** <50%<br>**LV hypertrophy** (≥225g for males, ≥163 g for females measured by echocardiography; >203.5g for males, >140.3g for females, measured by CMR)<br>**LA enlargement** – LA diameter ≥4.7cm for males, ≥4.3cm for females, where available.<br>**Abnormal biomarkers** - BNP ≥35pg/mL, NT-proBNP ≥125pg/mL or high-sensitivity cardiac troponin ≥99th percentile for the assay used.	43%

BNP, brain natriuretic peptide; GLS, global longitudinal strain; EF, ejection fraction; LA, left atrial; LV, left ventricle; NT-proBNP, N-terminal pro b-type natriuretic peptide.

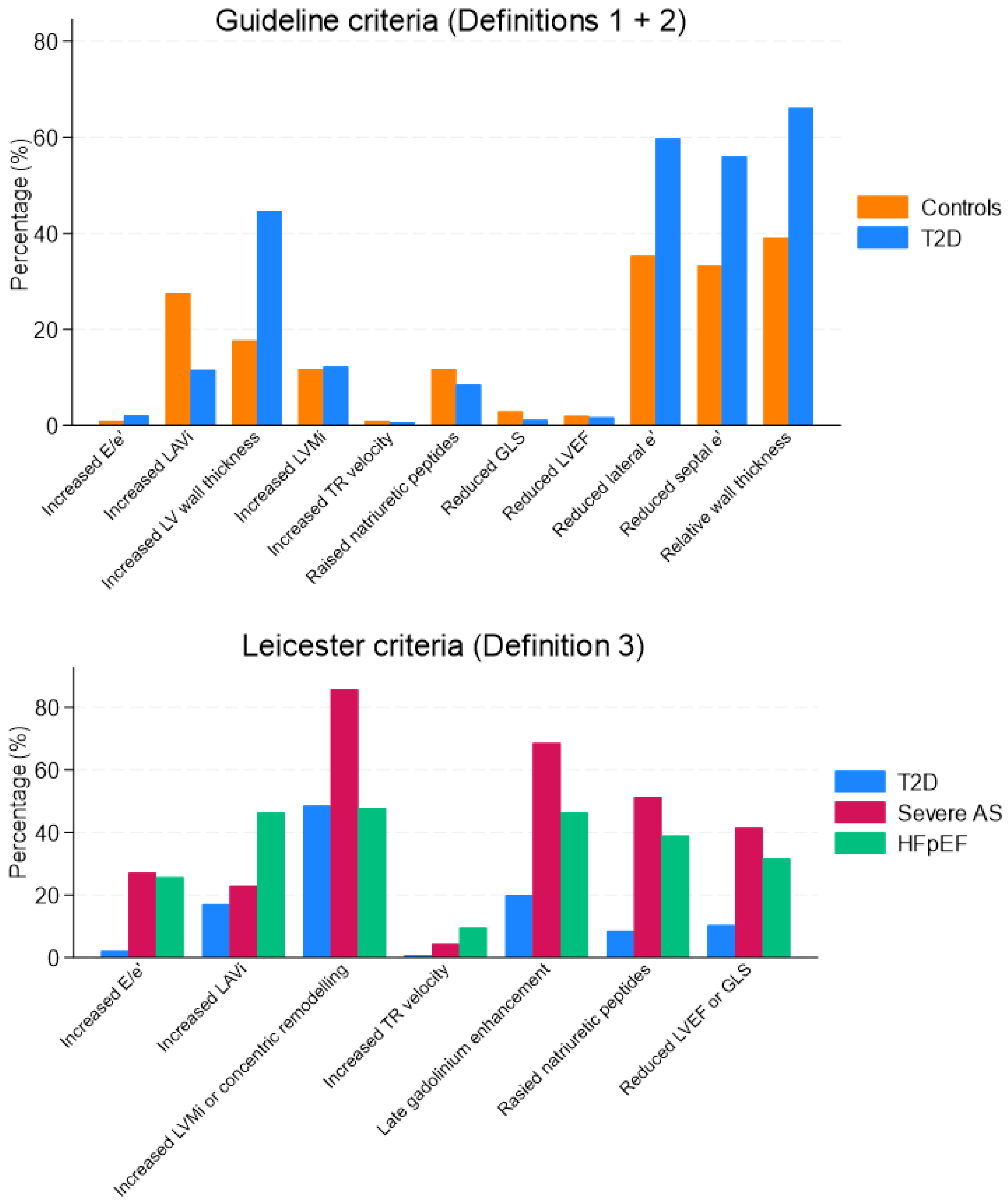

Figure S1. Prevalence of each imaging criterion for SBHF definitions, using their respective thresholds. GLS, global longitudinal strain; LAVI, left atrial volume indexed; LV, left ventricular; LVEF, left ventricular ejection fraction; LVMI, left ventricular mass indexed; RWT, relative wall thickness; TR, tricuspid regurgitation.

Reference

1. Kiencke S, Handschin R, von Dahlen R, Muser J, Brunner-Larocca HP, Schumann J, et al. Pre-clinical diabetic cardiomyopathy: prevalence, screening, and outcome. Eur J Heart Fail. 2010;12(9):951-7.
2. Wang Y, Yang H, Huynh Q, Nolan M, Negishi K, Marwick TH. Diagnosis of Nonischemic Stage B Heart Failure in Type 2 Diabetes Mellitus: Optimal Parameters for Prediction of Heart Failure. JACC Cardiovasc Imaging. 2018;11(10):1390-400.
3. Oo MM, Tan Chung Zhen I, Ng KS, Tan KL, Tan ATB, Vethakkan SR, et al. Observational study investigating the prevalence of asymptomatic stage B heart failure in patients with type 2 diabetes who are not known to have coronary artery disease. BMJ Open. 2021;11(1):e039869.
4. Mohebi R, Wang D, Lau ES, Parekh JK, Allen N, Psaty BM, et al. Effect of 2022 ACC/AHA/HFSA Criteria on Stages of Heart Failure in a Pooled Community Cohort. J Am Coll Cardiol. 2023;81(23):2231-42.
